# Correction to: Deep reinforcement learning for turbulent drag reduction in channel flows

**DOI:** 10.1140/epje/s10189-023-00304-8

**Published:** 2023-06-29

**Authors:** Luca Guastoni, Jean Rabault, Philipp Schlatter, Hossein Azizpour, Ricardo Vinuesa

**Affiliations:** 1 FLOW, Engineering Mechanics, KTH Royal Institute of Technology, 100 44 Stockholm, Sweden; 2 Swedish E-Science Research Centre (SeRC), 100 44 Stockholm, Sweden; 3 IT Department, Norwegian Meteorological Institute, Postboks 43, 0313 Oslo, Norway; 4 School of Electrical Engineering and Computer Science, KTH Royal Institute of Technology, 100 44 Stockholm, Sweden

**Correction to** **:** **Eur. Phys. J. E (2023) 46:27**

https://doi.org/10.1140/epje/s10189-023-00285-8

In this article the annotations have been missing for Fig. 1; the figure should have appeared as shown below. The original article has been corrected.

**Fig. 1:**
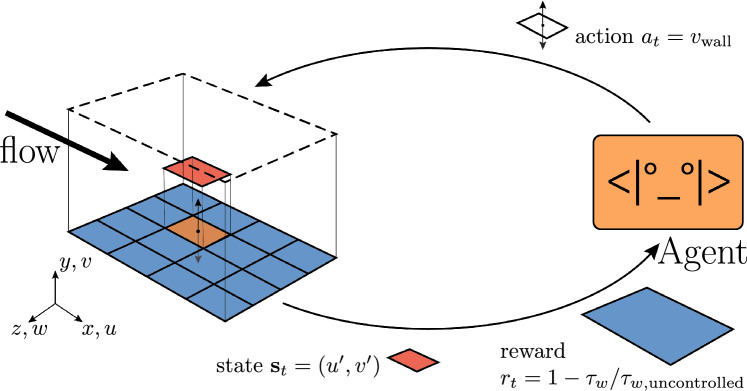
Overview of our multi-agent DRL approach to drag reduction. The simulation domain is shown on the left. The agents are organized in a grid $N_{\mathrm{{CTRLx}}} \times N_{\mathrm{{CTRLz}}}$. Each agents observes the velocity fluctuations in the streamwise (*u*′) and wall-normal (*v*′) direction. The reward is the percentage variation of the wall-shear stress $\tau _w$. Based on the state, each agent acts by imposing a wall-normal velocity *v* at the wall

